# The RAI-6 Questionnaire: A New Screening Questionnaire to Monitor Complications of Radioiodine Treatment

**DOI:** 10.3389/fsurg.2021.641945

**Published:** 2021-09-01

**Authors:** Samanta Buchholzer, Sethu Thakachy Subha, Livia Tchérémissinoff, Franciscus Boselie, Frédéric Triponez, Frédéric Faure, Jean-Michel Lopez, Urs Borner, Tobias Kleinjung, Jörg D. Seebach, Pavel Dulguerov, Francis Marchal

**Affiliations:** ^1^Department of Maxillofacial Surgery and Oral Medicine and Pathology, Geneva University Hospitals, Geneva, Switzerland; ^2^Department of Surgery/ENT, Universiti Putra Malaysia (UPM), Serdang, Malaysia; ^3^European Sialendoscopy Training Center, Geneva, Switzerland; ^4^Department of Otolaryngology-Head and Neck Surgery, University Hospitals of Geneva, Geneva, Switzerland; ^5^Department of Thoracic Surgery, University Hospitals of Geneva, Geneva, Switzerland; ^6^Department of Otolaryngology-Head and Neck Surgery, University Hospitals of Lyon, Lyon, France; ^7^Department of Otolaryngology-Head and Neck Surgery, Perpignan Hospital, Perpignan, France; ^8^Department of Otolaryngology-Head and Neck Surgery, University Hospitals of Bern, Bern, Switzerland; ^9^Department of Otolaryngology-Head and Neck Surgery, University Hospitals of Zürich, Zurich, Switzerland; ^10^Division of Allergy and Immunology, Department of Medicine, University Hospitals of Geneva, Geneva, Switzerland; ^11^Hôpital de la Tour, Geneva University, Geneva, Switzerland

**Keywords:** sialadenitis, radioiodine, xerostomia, xerophthalmia, questionnaire

## Abstract

**Objective:** The aim of this study is to develop a simple and efficient screening questionnaire to be able to routinely monitor potential radioiodine therapy-induced complications.

**Materials and Methods:** A new radioiodine 6 (RAI-6) questionnaire containing six questions adressing salivary, ocular, and nasal symptoms as well as quality of life was developed. Validation of the RAI-6 questionnaire was assessed with a group of fifty-four patients diagnosed with differentiated thyroid carcinoma treated post-operatively with radioiodine therapy, and in a group of fifty healthy volunteers. The patient's group was subdivided into subgroups according to the radioiodine dose received: 23 patients received less or 30 mCi, 28 patients received 100 mCi, and three patients received between 200 and 300 mCi. We asked the patients to complete the RAI-6 questionnaire in a retrospective manner, regarding their situation before radioiodine therapy and regarding their actual symptoms after radioiodine therapy. The time needed to complete the RAI-6 was also assessed both in patients and in healthy volunteers.

**Results:** The mean post radioiodine treatment RAI-6 score were significantly higher than the mean pre radioiodine RAI-6 scores (*p* < 0.001) and the scores of healthy participants (*p* < 0.001). The mean total RAI-6 scores increased significantly with increasing radioiodine dose. A total mean RAI-6 score of each question was also analysed and revealed that ocular and nasal discomfort as well as quality of life were the items which affected the patients most after radioiodine treatment. The mean time to fill the RAI-6 questionnaire was 2 min for patients and 49 s for healthy volunteers.

**Conclusion:** The RAI-6 represents a new questionnaire which is easy and quick to complete. This simple screening tool can be recommended for general clinical practise and further epidemiological research.

## Introduction

Thyroid carcinoma is the most common endocrine malignancy, ranking in ninth place for cancer incidence worldwide (1). The common histological types, papillary and follicular carcinoma, are broadly classified as well-differentiated thyroid carcinoma (2). Generally, a multimodality treatment approach is adopted for these tumours consisting of total thyroidectomy followed by radioiodine treatment (2, 3). The most common adverse effects of radioiodine treatment are xerostomia, sialadenitis, dry eyes, dry nose, and dysgeusia (4–7) with either acute onset after a therapeutic dose of radioactive iodine, or chronic onset with progressive intensity lasting over months or years. The incidence of acute sialadenitis is reported to be 24–67% whereas chronic sialadenitis occurs in 11 to 43% (4). The diagnosis of radioiodine-induced sialadenitis (RAIS) is clinical, essentially depending on the patient's complaints (5). Unfortunately, RAIS may be frequently missed because these symptoms are not investigated by clinicians, especially if they do not appear shortly after the radioiodine treatment. Xerostomia usually appears 3 months to 1 year after the radioiodine treatment in 15 to 54% of patients (8, 9). There are only a few retrospective studies regarding nasolacrimal complications of radioiodine therapy (6); Jonklass et al. reported that nasal and lacrimal dysfunction occurs in ~10% of patients, after a mean dose of 109 mCi of radioiodine. Even though salivary side effects of radioiodine decrease many years after the radioiodine treatment, Le Roux et al. still found a high rate of xerostomia (31.9%) 6 years after the radioiodine treatment, as well as 14% of their patients also experiencing salivary glands swellings (10). Patients still experiencing symptoms more than 1 year after the radioiodine treatment are at high risk to present permanent salivary and lacrymal glands damages (11). However, patients treated with low radioiodine dose (30 mCi or less) are very unlikely to experience long lasting complications [REF idem] (7). Patients are usually recommended to drink two to three litters of water every 24 h during the following days after the radioiodine (RAI) treatment to allow the radioiodine excretion of the salivary glands. Although investigated in the literature, the use of lemon juice to reduce potential complications has no effect and may even increase the damage to the salivary glands (12).

The high variability in the incidence of radioiodine complications, especially regarding xerostomia and sialadenitis, suggests a lack of standardisation regarding the methods used to evaluate these symptoms and a potential underestimation of these alterations in some studies. Also, regarding the literature no consensus evaluation method exist, as nine studies were found to use questionnaires to evaluate radioiodine complications; however each study used a different questionnaire, either custom-made or already existing xerostomia questionnaires (13–21). Therefore, we strongly believe that there is a need for a simple tool enabling a rapid assessment of radioiodine potential adverse effects pre- and post-radioiodine therapy allowing to compare the scores of each patient before and after treatment. Here, we present a new 6-item questionnaire, called RAI-6, as well as its validation with a retrospective cohort of thyroid carcinoma patients having received radioiodine therapy and a group of control healthy patients.

## Methods

The radioiodine 6 (RAI-6) is a six-item questionnaire using a visual analogue scale with a 0 to 10 scoring (Table 1), where “0” indicates the complete absence of symptoms and “10” indicates its maximum severity. The first three questions assess patient's subjective feeling of dryness of the mouth and discomfort of the eyes and nose. The next two questions concern the frequency of tightness and swelling of the salivary glands. The last question evaluates the quality of life in relation to those symptoms.

**Table 1:** RAI-6 questionnaire (0–10 visual analogue scale).

1. Evaluate the dryness of your mouth
(None) 0 1 2 3 4 5 6 7 8 9 10 (Unbearable)
2. Evaluate the discomfort of your eyes
(None) 0 1 2 3 4 5 6 7 8 9 10 (Unbearable)
3. Evaluate the discomfort of your nose
(None) 0 1 2 3 4 5 6 7 8 9 10 (Unbearable)
4. Evaluate the frequency of itching and/or tightness of your salivary glands (in front of the ears/below the lower jaw)
Never 0 1 2 3 4 5 6 7 8 9 10 (continuous)
5. Evaluate the frequency of swelling of your salivary glands (in front of the ears/below the lower jaw)
(Never) 0 1 2 3 4 5 6 7 8 9 10 (continuous)
6. Evaluate your overall quality of life related to those symptoms
(No impact) 0 1 2 3 4 5 6 7 8 9 10 (severe impact)

The 6 items of the RAI-6 were based on the radioiodine complications described in the literature (4–7).

A retrospective study was conducted after obtaining the ethical approval from the institutional ethical committee (reference number 2019-00253). The study consisted of two groups of participants: the first group included adult patients with well-differentiated thyroid carcinoma already treated with radioactive iodine therapy and the second group was composed of healthy volunteers. Informed consent was obtained from all participants. The group of patients already treated with radioactive iodine were asked to complete the RAI-6 regarding their actual symptoms and also regarding their initial state, before undergoing the radioiodine treatment. The mean time between radioiodine treatment and the assessment of the RAI-6 questionnaire was 3.6 years (±1.4 years). Participants previously treated with chemotherapy and/or radiotherapy for other head and neck cancers, as well as those presenting xerostomia due to systemic disorders such as Sjögren's syndrome or linked to medications (c.f. anticholinergics, antihistamines) were excluded from the study.

The patients were contacted to complete the questionnaire either by phone or during an out-patient consultation. The time needed to complete the questionnaire and the score results were assessed and compared for statistical significance using a two-tailed Fisher F test (Microsoft Excel 365 with XLSTAT365 add-on) between patients pre- and post-radioiodine treatment and between the two study groups.

## Results

The results regarding the participants demographics and time to complete the RAI-6 are detailed in Table 2. Fifty healthy volunteers and 54 patients fulfilled the inclusion criteria were included in our study. In the patient's group, 33 patients (61.1%) were females and 21 (32%) were males. The age varied from 30 to 90 years with a mean of 56.2 years. The time needed to complete the RAI-6 questionnaire amounted to 2 min (50–180”). Most of the patients underwent total thyroidectomy for papillary carcinoma (41/54 patients, 76%), follicular carcinoma (5/54 patients, 9.2%) or undifferentiated thyroid carcinoma (1/54 patients, 1.8%). Seven other patients (13%) underwent radioiodine therapy either for Gravers diseases, or for an autonomous nodule accompanied by hyperthyroidism. Radioactive iodine therapy was administered with a dose varying from 4.86 to 300 millicuries (mCi). Twenty-three patients received 30 mCi or less, 28 patients received 100 mCi, and three patients received between 200 and 300 mCi. The mean total RAI-6 score amounted 1.7 before radioiodine treatment and 7.6 after radioiodine treatment, a statistically significant difference (*p* < 0.001) (Table 3).

**Table 2:** Participants demographics and time to complete RAI-6.

	**Healthy volunteers**	**Patients (radioiodine treatment)**
Total (*n*)	50	54
Age	37.98	56.2
Male	16 (32%)	21 (38.9%)
Female	34 (68%)	33 (61.1%)
Papillary thyroid carcinoma	0	41 (76%)
Follicular thyroid carcinoma	0	5 (9.2%)
Undifferentiated thyroid carcinoma	0	1 (1.8%)
Other (Gravers Disease, autonomous nodule with hyperthyreoditis)	0	7 (13%)
RAI: time to complete (seconds)	49 s (12–120 s)	120 s (50–180 s)

**Table 3:** Healthy volunteers RAI-6 scores and pre- and post-radioiodine therapy patient's mean RAI-6 scores according to the radioiodine dose received.

	**Number of patients**	**mCi**	**Mean pre radioiodine treatment RAI-6 total score**	**Mean post radioiodine treatment RAI-6 total score**	***p*-value (comparison of mean RAI-6 total scores between pre- and post-radioiodine treatment)**
Healthy	50	0	1.16	–	
Patients	54	All	1.7	7.6	<0.001
Patients	23	< or = 30	1.32	7.48	<0.001
Patients	28	100	1.8	6.4	<0.001
Patients	3	200–300	3.7	19.2	0.319

The 23 patients receiving 30 mCi or less had a RAI-6 total mean score of 1.32 prior to-radioiodine and 7.48 after radioiodine therapy (*p* < 0.001). In the sub-group who received 100 mCi of radioiodine dose the pre- and post-treatment mean RAI-6 total score were 1.8 and 6.4, respectively (*p* < 0.001). Finally, the three patients receiving between 200 and 300 mCi, the mean RAI-6 total score was 3.7 before and 19.2 after radioiodine therapy (*p* = 0.32) (Table 3).

The mean pre- and post-radioiodine treatment RAI-6 scores were also analysed separately for each item (Q1, Q2, Q3, Q4+Q5, and Q6) regarding every subgroup of patients depending on their radioiodine dose (< or = 30, 100, 200–300 mCi) and the results are detailed in Tables 4–6. The items 5 and 6 were analysed together as they both assess sialadenitis symptoms. Items regarding ocular and nasal dryness as well as quality of life were those who were mostly worsened by radioiodine therapy, increasing with the dose.

**Table 4:** Pre- and post-operative mean RAI-6 scores in patients who received< or = 30 mCi.

**Patients treated with ≤ 30 mCi (*n* = 23)**	**Mean pre radioiodine treatment RAI-6 score**	**Mean post radioiodine treatment RAI-6 score**
Q1	0.22	1.8
Q2	0.5	2.2
Q3	0.3	1.74
Q4 + Q5	0.2	0.54
Q6	0.1	1.2

**Table 5:** Pre- and post-operative mean RAI-6 scores in patients who received 100 mCi.

**Patients treated with 100 mCi (*n* = 28)**	**Mean pre radioiodine treatment RAI-6 score**	**Mean post radioiodine treatment RAI-6 score**
Q1	0.5	1.1
Q2	0.7	2.3
Q3	0.4	1.5
Q4 + Q5	0.1	0.5
Q6	0.1	1

**Table 6:** Pre- and post-operative mean RAI-6 scores in patients who received 200–300 mCi.

**Patients treated with 200–300 mCi (*n* = 3)**	**Mean pre radioiodine treatment RAI-6 score**	**Mean post radioiodine treatment RAI-6 score**
Q1	1	1
Q2	0.7	3.3
Q3	2	7.3
Q4 + Q5	0	2.3
Q6	0	5.3

Regarding the group of healthy volunteers, the mean age was 38 years old and 34 patients (68%) were females and 16 (32%) were males. The mean time for healthy volunteers to complete the RAI-6 was 49 s (12–120”) (Table 2 None of the healthy participants addressed sialadenitis symptoms. The mean total score for every other item remained under 0.5 (Table 7). The mean total RAI-6 score for healthy participants significantly differed from the post-treatment RAI-6 values of patients who underwent radioiodine therapy (*p* < 0.001).

**Table 7:** Mean RAI-6 scores in healthy participants.

**Healthy**	**Mean RAI-6 total score**
Q1	0.22
Q2	0.32
Q3	0.34
Q4 + Q5	0
Q6	0.28

## Discussion

Regarding the literature, 10 studies used questionnaires to evaluate the complications of radioiodine therapy. Seven of these studies utilised a custom-made questionnaire. Regarding the last three studies, Bhayani et al. (19) and Klein Hesselink et al. (20) used the Quality-of-Life Xerostomia Questionnaire (QOLXQ) and the Xerostomia Inventory, respectively. The last study by Hollingsworth et al. (21) used modified questions from another questionnaire previously validated with patients who underwent external beam radiation therapy.

Our new screening questionnaire RAI-6 attempts to assess the complications of radioiodine therapy, especially salivary gland dysfunction, oral dryness, ocular and nasal discomfort, and their influence on the quality of life. To validate the RAI-6 questionnaire we compared the total scores between healthy participants and patients who underwent radioiodine therapy as well as the scores pre- and post-radioiodine therapy between those same patients. Moreover, the time to complete the RAI-6 questionnaire was also assessed and compared between healthy volunteers and patients who had undergone radioiodine treatment.

The total mean RAI-6 score was significantly higher in patients treated with radioiodine (7.6) compared to healthy volunteers (1.16). Also, the mean RAI-6 scores were higher in patients after their radioiodine treatment (7.6) compared to their RAI-6 mean total scores before RAI-6 therapy (1.7). The total mean RAI-6 score of patients before radioiodine therapy is slightly higher (1.7) than the one of healthy participants (1.16). We attribute this discrepancy to the high difference of the mean ages between these two groups as the patient's group was almost 20 years older.

Increasingly higher mean RAI-6 scores are found when the administered radioiodine dose is 200–300 mCi (19.2) in comparison to a dose of < or = to 30 mCi (7.48). Given the assumption that the higher radioiodine doses are associated with an increased symptoms, we can conclude that the RAI-6 questionnaire assesses well the severity of symptoms. However, the RAI-6 scores of patients who received 100 mCi (6.4) are lower than those of patients who received 30 mCi (7.48). We believe that this result may be biassed, and linked to the retrospective nature of our study and the limited number of patients.

Regarding the first RAI-6 question evaluating xerostomia, the post radioiodine mean score of patients interestingly increased in a higher manner in patients who received lower radioiodine doses compared to those who received higher radioiodine doses. No rational explanation can be deducted, although this result may be linked to the bias of our limited number of patients.

The second question, by addressing ocular discomfort in a general manner, aims to include several ocular symptoms such as epiphora, xerophthalmia, and recurrent or chronic conjunctivitis. Ocular dryness is a relatively frequent adverse effect of radioiodine therapy and its' associated problems tend to persist for a prolonged period after the end of the treatment (22).

The mean post radioiodine treatment RAI-6 scores regarding the second question were higher than the pre-treatment mean RAI-6 scores and this difference increased with the dose.

The third question also aims to assess nasal discomfort in a general manner to include nasal dryness, pain, sores and epistaxis. The mean post radioiodine treatment RAI-6 scores regarding nasal discomfort were also significantly higher than the mean RAI-6 scores before radioiodine treatment and this difference increased with the dose.

Ocular et nasal discomfort appear to be highly rated in our patient's group after radioiodine therapy. However, only few studies describe ocular (13, 17) and nasal side effects of radioiodine therapy (16, 17) which may result into an underestimated rate of those complications.

The fourth and fifth questions aim to assess sialadenitis symptoms by evaluation itching and swelling of the salivary glands. The mean post radioiodine treatment RAI-6 scores regarding those two items was significantly higher than before treatment and this difference also increased with the radioiodine dose. Our results correlate with Dingle et al.who determined the prevalence of sialadenitis in patients treated with radioiodine for thyroid cancer using a 14 items self-administered questionnaire (15) and concluded that the prevalence of sialadenitis was 26% for patients who received between 100 and 200 mCi of radioiodine and 43% for patients who were treated with over 200 mCi of radioiodine. It is interesting to notice that sialadenitis did not occur in patients who received <100 mCi of radioiodine. The overall reported prevalence of sialadenitis following radioiodine therapy varies from 2 to 67% in the literature (5), which would imply an underestimated rate from many studies.

The last question evaluates the adverse effects of radioiodine therapy on the quality of life of patients, which was rarely assessed in previous studies. Dingle et al.evaluated the quality of life with the University of Washington Quality of Life Questionnaire (UW-QOL), and the Xerostomia-Related Quality of Life Scale (Xe QOLS) (15), however other studies did not include direct quality of life items (18). Bhayani et al.used a standard quality of life (QOL) questionnaire and reported durable improvement in symptoms after sialendoscopy (19). Due to the high survival rate, and the likelihood of long-term complications after radioiodine therapy, QOL needs to be evaluated in clinical practise (23). Indeed, QOL is an important indicator to identify patients requiring further care. In our study QOL was affected after radioiodine treatment, especially with doses over or equal to 200 mCi.

The mean age of our patients was 56.2 years with a female predilection (61.1%), which is in accordance with the previous studies (24–26). Hollingsworth et al., showed that increased age and female gender might be risk factors for radioiodine induced salivary gland damage in thyroid cancer patients (21). Statistical analysis of our study data demonstrated that a worse score on the RAI-6 questionnaire does correlated with the radioiodine dose given. Our study results are comparable with the studies by Almeida et al. (14), Krcalova et al. (27), and Fard-Esfahani et al. (28). The study by Almeida et al. showed that doses higher than 150 mCi have more side effects on salivary glands (13). Krcalova et al.concluded that RAI therapy up to 5.55 GBq (150 mCi) does not substantially decrease saliva production (27). Fard-Esfahani et al. suggested that RAIS is more frequent in differentiated thyroid cancer patients, with the higher dose of 100 mCi, compared to patients treated for hyperthyroidism with doses <30 mCi (28).

Regarding healthy participants, none of them presented with sialadenitis symptoms, however some of them experienced low severity xerostomia, ocular and nasal discomfort symptoms which slightly affected their QOL. It is therefore interesting to notice that even healthy volunteers who do not present any risk factors still may present oral dryness, ocular and nasal discomfort from unknown aetiologies.

The purpose of the RAI is to be a screening test which aims to be quickly completed. If the RAI shows any symptoms, the recently developed MSGS questionnaire should be completed to assess more precisely symptoms regarding salivary glands, as well as clinical and/or radiological complementary investigations adapted to every patient at the discretion of the clinician. The RAI-6 questionnaire evaluates symptoms using a visual analogue scale (VAS) system considered as a gold standard for the assessment of clinical symptoms (29). The mean time necessary to complete the RAI-6 questionnaire was 2 min for patients and 49 s for healthy volunteers which makes it little time consuming. None of the previous reports on questionnaires regarding the complications of radioiodine therapy assessed the time needed for their completion. However, most of these questionnaires had more items than the present one; Choi et al. (16) and Jonklaas et al. (17) used questionnaires that comprised 15 to 21 items, which is in our opinion not appropriate for a screening score which aims to be rapidly completed. Our questionnaire is designed to focus on the most salient and subjective aspects, using only six easily understandable and clinically relevant questions, covering the most common complications of radioiodine therapy, and their impact on the quality of life. At the same time, this questionnaire does not impose a burden on patients or clinicians, due to its short format.

The limitations of our study are the small sample size and the retrospective evaluation of the patients who underwent radioiodine therapy. In fact, patients who underwent radioiodine therapy were asked to complete the RAI-6 questionnaire 3.6 years after the end of their treatment and to estimate if any eventual symptoms were also present before radioiodine treatment. A prospective study including patients who complete the RAI-6 questionnaire before and after radioiodine therapy at specific timelines would allow a more accurate understanding of radioiodine adverse effects.

## Conclusion

This preliminary study highlights the advantages of the newly developed RAI-6 questionnaire to access complications of radioiodine therapy. Our validation results indicate that the RAI-6 questionnaire manages to quickly and concisely identify the main complications of RAI therapy: sialadenitis, xerostomia, ocular, and nasal discomfort. We also found that radioiodine-induced complications have a significant impact on the quality of life.

## Data Availability Statement

The original contributions presented in the study are included in the article/supplementary material, further inquiries can be directed to the corresponding author/s.

## Ethics Statement

The studies involving human participants were reviewed and approved by Commission cantonale d'Ethique de la Recherche sur l'être humain (CCER) Geneva: reference number 2019-00253. The patients/participants provided their written informed consent to participate in this study.

## Author Contributions

SB, ST, FB, PD, FM, and JS contributed to the redaction of the manuscript. All other co-authors proceeded with the patient's consultations and participated to the improvement of the radioiodine 6 questionnaire.

## Conflict of Interest

The authors declare that the research was conducted in the absence of any commercial or financial relationships that could be construed as a potential conflict of interest.

## Publisher's Note

All claims expressed in this article are solely those of the authors and do not necessarily represent those of their affiliated organizations, or those of the publisher, the editors and the reviewers. Any product that may be evaluated in this article, or claim that may be made by its manufacturer, is not guaranteed or endorsed by the publisher.

## References

[1] Siegel R Miller KD Jemal A. Cancer statistics, 2019. CA Cancer J Clin. (2019) 69:7–34. 10.3322/caac.21551 30620402

[2] Ko KY Kao CH Lin CL Huang WS Yen RF. (131)I treatment for thyroid cancer and the risk of developing salivary and lacrimal gland dysfunction and a second primary malignancy: a nationwide population-based cohort study. Eur J Nucl Med Mol Imaging. (2015) 42:1172–8. 10.1007/s00259-015-3055-0 25900274

[3] Chen AY Jemal A Ward EM. Increasing incidence of differentiated thyroid cancer in the United States, 1988–2005. Cancer. (2009) 115:3801–7. 10.1002/cncr.24416 19598221

[4] Silberstein EB. Reducing the incidence of 131I-induced sialadenitis: the role of pilocarpine. J Nucl Med. (2008) 49:546–49. 10.2967/jnumed.107.049411 18344428

[5] Nostrand DV. Sialoadenitis secondary to 131I therapy for well differentiated thyroid cancer. Oral Dis. (2011) 17:154–61. 10.1111/j.1601-0825.2010.01726.x 21029259

[6] Clement SC Peeters RP Ronckers CM Links TP van den Heuvel-Eibrink MM Nieveen van Dijkum EJ. Intermediate and long-term adverse effects of radioiodine therapy for differentiated thyroid carcinoma–a systematic review. Cancer Treat Rev. (2015) 41:925–34. 10.1016/j.ctrv.2015.09.001 26421813

[7] Jonklaas J. Nasal symptoms after radioiodine therapy: a rarely described side effect with similar frequency to lacrimal dysfunction. Thyroid. (2014) 24:1806–14. 10.1089/thy.2014.0162 25090584 PMC4267770

[8] Caglar M Tuncel M Alpar R. Scintigraphic evaluation of salivary gland dysfunction in patients with thyroid cancer after radioiodine treatment. Clin Nucl Med. (2002) 27:767–71. 10.1097/00003072-200211000-00003 12394122

[9] Maruoka Y. A functional scoring system based on salivary gland scintigraphy for evaluating salivary gland dysfunction secondary to 131I therapy in patients with differentiated thyroid carcinoma. J Clin Diagn Res. (2017) 11:TC23–8. 10.7860/JCDR/2017/27340.10431 28969240 PMC5620881

[10] Le Roux MK Graillon N Guyot L Taieb D Galli P Godio-Raboutet Y. Salivary side effects after radioiodine treatment for differentiated papillary thyroid carcinoma: long-term study. Head Neck. (2020) 42:3133–40. 10.1002/hed.26359 32652742

[11] Rosario PW Calsolari MR. Salivary and lacrimal gland dysfunction after remnant ablation with radioactive iodine in patients with differentiated thyroid carcinoma prepared with recombinant human thyrotropin. Thyroid. (2013) 23:617–9. 10.1089/thy.2012.0050 23136908

[12] Nakada K Ishibashi T Takei T Hirata K Shinohara K Katoh S. Does lemon candy decrease salivary gland damage after radioiodine therapy for thyroid cancer? J Nucl Med. (2005) 46:261–6. 15695785

[13] Alexander C Bader JB Schaefer A Finke C Kirsch CM. Intermediate and long-term side effects of high-dose radioiodine therapy for thyroid carcinoma. J Nucl Med. (1998) 39:1551–4. 9744341

[14] Almeida JP Sanabria AE Lima EN Kowalski LP. Late side effects of radioactive iodine on salivary gland function in patients with thyroid cancer. Head Neck. (2011) 33:686–90. 10.1002/hed.21520 21484917

[15] Dingle IF Mishoe AE Nguyen SA Overton LJ Gillespie MB. Salivary morbidity and quality of life following radioactive iodine for well-differentiated thyroid cancer. Otolaryngol Head Neck Surg. (2013) 148:746–52. 10.1177/0194599813479777 23462656

[16] Choi JS Hong SB Hyun IY Lim JY Kim YM. Effects of salivary secretion stimulation on the treatment of chronic radioactive iodine-induced sialadenitis. Thyroid. (2015) 25:839–45. 10.1089/thy.2014.0525. 25809840

[17] Jonklaas J Wang H Esposito G. Salivary function after radioiodine therapy: poor correlation between symptoms and salivary scintigraphy. Front Endocrinology. (2015) 6:100. 10.3389/fendo.2015.00100 26136724 PMC4470264

[18] Moreddu E Baumstarck-Barrau K Gabriel S Fakhry N Sebag F Mundler O. Incidence of salivary side effects after radioiodine treatment using a new specifically-designed questionnaire. Br J Oral Maxillofac Surg. (2017) 55:609–612. 10.1016/j.bjoms.2017.03.019 28456449

[19] Bhayani MK Acharya V Kongkiatkamon S Farah S Roberts DB Sterba J. Sialendoscopy for patients with radioiodine-induced sialadenitis and xerostomia. Thyroid. (2015) 25:834–8. 10.1089/thy.2014.0572 25860842 PMC5118964

[20] Klein Hesselink EN Brouwers AH de Jong JR van der Horst-Schrivers AN Coppes RP Lefrandt JD. Effects of radioiodine treatment on salivary gland function in patients with differentiated thyroid carcinoma: A prospective study. J Nucl Med. (2016) 57:1685–91. 10.2967/jnumed.115.169888 27339871

[21] Hollingsworth B Senter L Zhang X Brock GN Jarjour W Nagy R. Risk factors of ^131^I-induced salivary gland damage in thyroid cancer patients. J Clin Endocrinol Metab. (2016) 101:4085–93. 10.1210/jc.2016-1605 27533304 PMC5095242

[22] Zettinig G Hanselmayer G Fueger BJ Hofmann A Pirich C Nepp J. Long-term impairment of the lacrimal glands after radioiodine therapy: a cross-sectional study. Eur J Nucl Med Mol Imaging. (2002) 29:1428–32. 10.1007/s00259-002-0969-0 12397459

[23] Bãrbuş E Peştean C Larg MI Piciu D. Quality of life in thyroid cancer patients: a literature review. Clujul Med. (2017) 90:147–53. 10.15386/cjmed-703 28559697 PMC5433565

[24] Rahbari R Zhang L Kebebew E. Thyroid cancer gender disparity. Fut Oncol. (2010) 6:1771–9. 10.2217/fon.10.127 21142662 PMC3077966

[25] Yan H Pang P Wang F Tian W Luo YK Huang W. Dynamic profile of differentiated thyroid cancer in male and female patients with thyroidectomy during 2000–2013 in China: a retrospective study. Sci Rep. (2017) 7:15832. 10.1038/s41598-017-14963-z 29158505 PMC5696456

[26] Kansakar E Chang YJ Mehrabi M Mittal V. Expression of oestrogen receptor, progesterone receptor, and vascular endothelial growth factor-A in thyroid cancer. Am Surg. (2009) 75:785–9. 19774949

[27] Krcalova E Horacek J Gabalec F Zak P Dolezal J. Salivary gland function in thyroid cancer patients with radioiodine administration history. Biomed Pap Med Fac Univ Palacky Olomouc Czech Repub. (2019) 164:277–83. 10.5507/bp.2019.023 31223135

[28] Fard-Esfahani A Fallahi B Karimi M Beiki D Saghari M Emami-Ardekani A. Changes in salivary gland function following radioiodine therapy of thyroid diseases: a comparison of high-dose therapy for thyroid cancer and low-dose therapy for benign thyroid disease. Iranian J Nucl Med. (2015) 23:1–7.

[29] Yarnitsky D Sprecher E Zaslansky RJA. Multiple session experimental pain measurement. Pain. (1996) 67:327–33. 10.1016/0304-3959(96)03110-7 8951926

